# Unusual Metastatic Sites in Malignant Phyllodes Tumor Detected on FDG PET/CT

**DOI:** 10.1055/s-0044-1786519

**Published:** 2024-04-25

**Authors:** Wai Ip Li, Koon Kiu Ng, Ting Kun Au Yong, Boom Ting Kung

**Affiliations:** 1 Nuclear Medicine Unit, Department of Diagnostic and Interventional Radiology, Queen Elizabeth Hospital, Hong Kong

**Keywords:** FDG, PET/CT, breast neoplasms, malignant, phyllodes tumors, metastases

## Abstract

Phyllodes tumor is a rare fibroepithelial neoplasm of the breast. This tumor tends to spread by hematogenous route, with common metastatic sites in the lungs, bones, and liver. Metastases to the pleura, stomach, pancreas, kidneys, and adrenal gland are rare. We present a case of a 52-year-old lady with malignant phyllodes tumor of breast undergone local tumor resection, followed by solitary lung metastasis with lobectomy, and subsequently diagnosed of multiple new metastatic sites in pleura, stomach, pancreas, kidneys, adrenal gland, and bone detected on 2-deoxy-2-[18F]fluoro-D-glucose positron emission tomography/computed tomography within 2 years.

## Introduction

Phyllodes tumors, rare fibroepithelial breast neoplasms, account for less than 1% of all breast tumors. These tumors exhibit a biphasic growth pattern, comprising stromal and epithelial components. 1 Classification of phyllodes tumors into benign, borderline, and malignant subtypes, proposed by the World Health Organization, is based on histopathological features such as stromal cellularity, mitotic activity, and tumor margins. 2 While most phyllodes tumors are benign, approximately 10 to 20% are malignant and have the potential to metastasize. 1

Malignant phyllodes tumors demonstrate aggressive behavior, local recurrence, and distant metastasis, with the lungs, bones, and liver being the most common sites of metastasis. 3 The malignant phyllodes tumor of breast shows risk of local recurrence up to 27% and distant metastasis up to 22%. 4 Metastases to unusual locations are extremely rare, but they have been reported in literatures. Here, we present a rare case of a 52-year-old woman with recurrent metastatic phyllodes tumor of the breast, exhibiting unusual metastatic sites on 2-deoxy-2-[18F]fluoro-D-glucose positron emission tomography/computed tomography (FDG PET/CT) imaging, including the lung, pleura, stomach, pancreas, kidneys, adrenal gland, and bone.

## Case Report

**Fig. 1:**
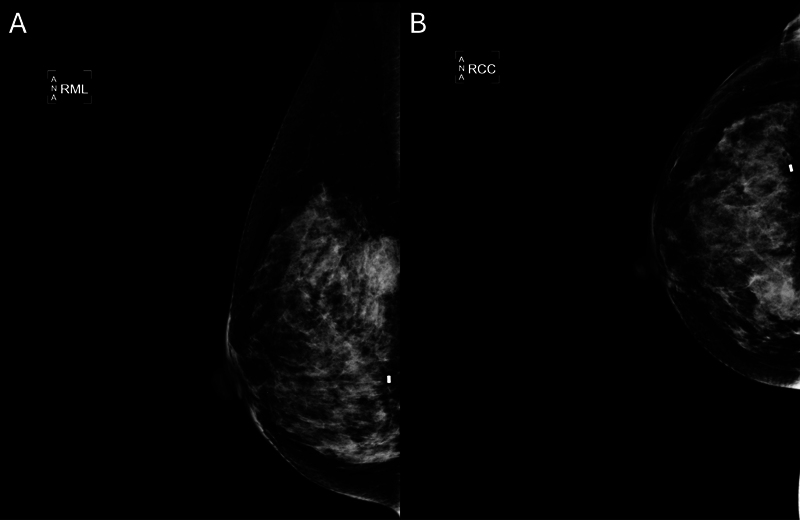
Mammography of right breast in mediolateral view ( **A** ) and craniocaudal view ( **B** ) showed a heterogeneously dense breast. An irregular spiculated high-density mass in the right upper inner quadrant was confirmed to be malignant phyllodes tumor by ultrasound-guided biopsy.

**Fig. 2:**
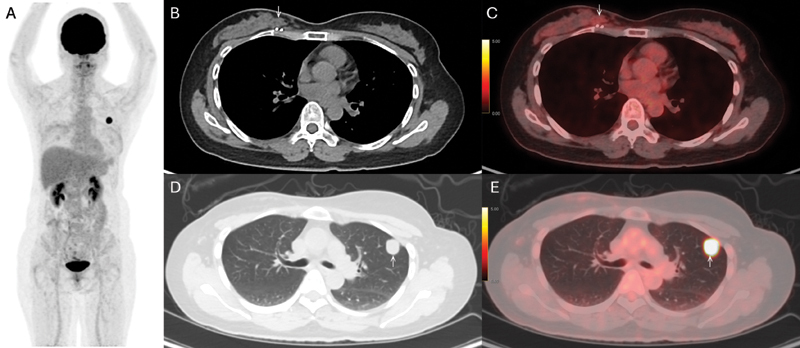
2-deoxy-2-[18F]fluoro-D-glucose positron emission tomography/computed tomography (FDG PET/CT) performed 7 months after lumpectomy of malignant phyllodes tumor of right breast. ( **A** ) Maximal intensity projection showed solitary lesion in left thorax. ( **B** and **C** ) Plain CT and fusion images revealed no discrete focal uptake in the lumpectomy site (white arrow) to suggest local recurrence. ( **D** and **E** ) Plain CT and fusion images showed solidary FDG-avid lung nodule at left upper lobe (white arrow) measuring standardized uptake value of 13.7 and 1.7 cm in diameter. Left upper lobe lobectomy was done 6 months later and confirmed it to be lung metastasis from malignant phyllodes tumor.

A 52-year-old lady, presented with progressive right breast mass for 2 months, subsequently assessed with mammography ( Fig. 1 ) and confirmed to be malignant phyllodes tumor by ultrasound-guided biopsy. She was treated with lumpectomy with an inadequate deep margin of 0.5 cm. She refused adjuvant radiotherapy to the right breast. A follow-up FDG PET/CT revealed no local recurrence in the right breast but a solitary FDG-avid (standardized uptake value of 13.7) left upper lobe lung nodule 7 months after the surgery ( Fig. 2 ). Left upper lobe lobectomy was done 6 months after the PET/CT, which confirmed to be metastatic phyllodes tumor with close margin to the chest wall, and thereafter received radiotherapy to the left anterior chest wall.

**Fig. 3:**
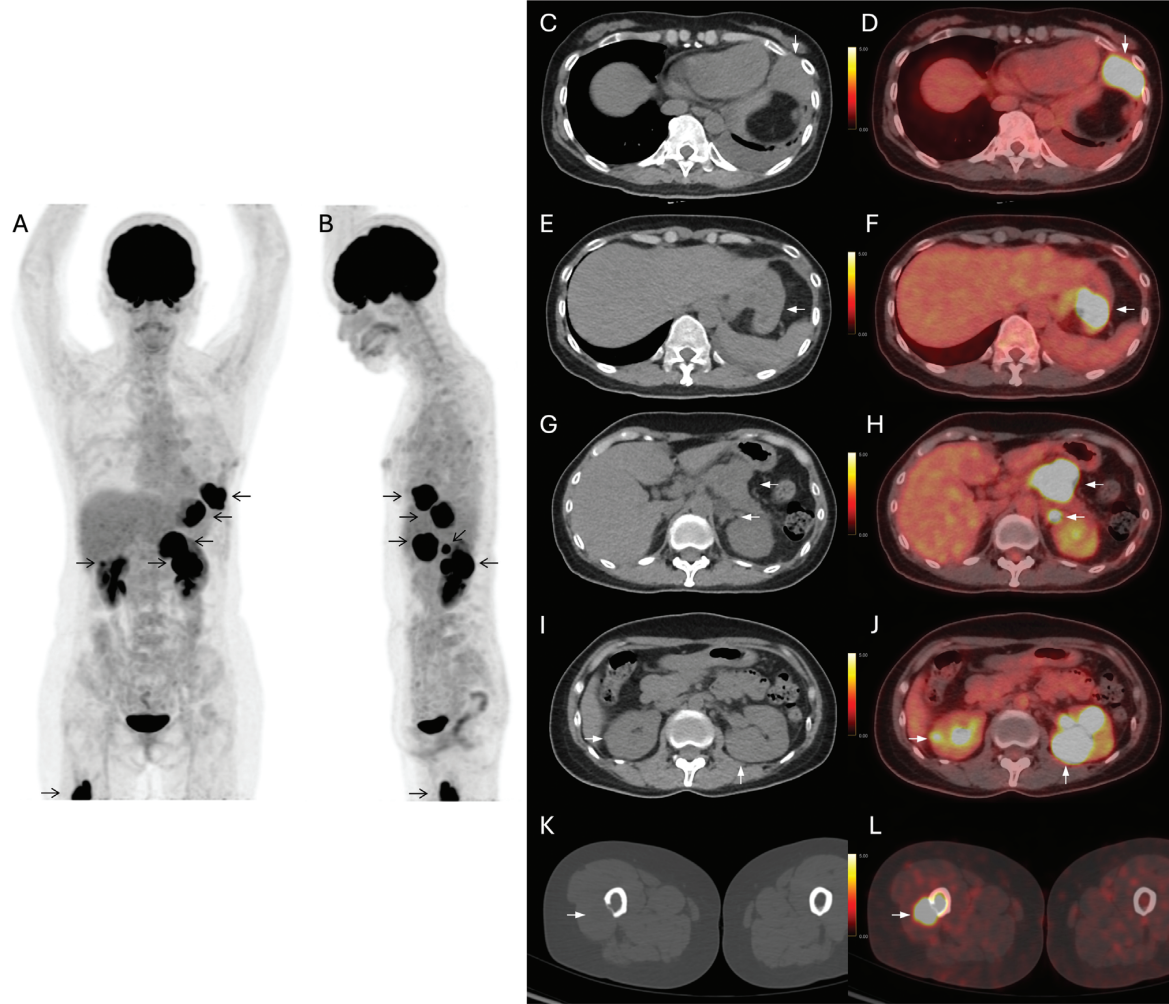
2-deoxy-2-[18F]fluoro-D-glucose positron emission tomography/computed tomography (FDG PET/CT) performed 7 months after lobectomy of left upper lobe of lung for surveillance. ( **A** and **B** ) Maximal intensity projection (MIP) of FDG PET/CT showed multiple new FDG-avid lesions in the left lower thorax, abdomen, and right lower limb. ( **C** and **D** ) Plain CT and axial fusion image revealed new FDG-avid left lower pleural thickening, compatible with pleural metastasis. ( **E** and **F** ) Plain CT and axial fusion image revealed a new FDG-avid gastric mass, compatible with gastric metastasis, which was confirmed by endoscopic guided biopsy. ( **G** and **H** ) Plain CT and axial fusion revealed new FDG-avid lesions in the pancreatic body and left adrenal gland, compatible with pancreatic and left adrenal metastases. ( **I** and **J** ) Plain CT and axial fusion revealed new FDG-avid bilateral renal masses, compatible with metastases. ( **K** and **L** ) Plain CT and axial fusion image revealed a new FDG-avid lytic bone lesion with soft tissue mass at proximal right femur, which was biopsy proven to be metastasis from malignant phyllodes tumor after pathological fracture with operation done 2 months after this scan. (Lesions as pointed out by black arrows on MIP images and white arrows on fusion images.)

Seven months after the lung surgery, a follow-up FDG PET/CT was performed for surveillance, showing multiple new FDG-avid lesions at the left pleura, stomach, pancreas, left adrenal gland, bilateral kidneys, and proximal right femur ( Fig. 3 ). Esophagogastroscopy revealed a neoplastic mass at the proximal gastric body with contact bleeding, which was confirmed to be a biopsy proven metastatic phyllodes tumor. Two months later, this lady was admitted to the hospital for a pathological right femoral fracture, with close reduction and internal fixation done. Intraoperational biopsy at the fracture site was conducted, revealing malignant spindle cell tumor, in keeping with metastasis from malignant phyllodes tumor. Considering the clinical context, other new FDG-avid lesions detected by the latest FDG PET/CT, including the left pleura, pancreas, left adrenal gland, and bilateral kidneys, were considered metastases from malignant phyllodes tumor of breast. She succumbed 2 months after the follow-up FDG PET/CT scan to systemic infection.

## Discussion

Phyllodes tumor of breast is a rare fibroepithelial breast cancer with unpredictable biologic behavior. Less aggressive form happens in most patients, which behave like benign fibroadenomas, while 10 to 20% are malignant, which progress in size locally and have the potential to metastasize. 5 A triple assessment is the standard diagnostic pathway for a breast mass, including clinical examination, imaging, and histology. The usual presentations of malignant phyllodes tumors are rapidly progressive breast mass that is mobile and nonpainful, and palpable nodal axillary metastasis in locally advanced disease is rare. 3 National Comprehensive Cancer Network (NCCN) suggested ultrasound and mammography for initial workup and regular surveillance for borderline and malignant phyllodes tumor. 6 Chest imaging including X-ray and computed tomography was recommended for patients with locally recurrent disease. 6 While core biopsy is a more sensitive technique, neither fine needle aspiration nor core biopsy can always distinguish phyllodes tumors from fibroadenomas. A definite diagnosis may require excision of the mass. 6 7 Surgical resection remains the mainstay of treatment approach, with wide local excision and mastectomy showing similar overall survival and recurrence. 3 Radiotherapy may reduce local recurrence rate, but there is no overall survival benefit. 3 In patients with metastatic disease, the prognosis is poor and regimen of chemotherapy follows NCCN soft tissue sarcoma guidelines. 3 6

Axillary nodal metastasis is rare for malignant phyllodes tumor but distant metastasis occurs in up to 22% of patients. 4 The most common sites of metastasis from malignant phyllodes tumors are lungs, bones, and liver, 3 while other sites of metastases have been reported in multiple organs, such as the heart, 8 9 pancreas, 8 10 gallbladder, 10 stomach, 11 duodenum, 12 small bowel, 13 adrenals, 14 kidneys, 15 and ovaries. 16 Conventional computed tomography and magnetic resonance imaging suffer from a limited scope of image to a dedicated body part of interest. FDG PET/CT, with the high sensitivity of malignant tumor detection and a comprehensive whole body image field of view, may be beneficial to high-risk patients to look for unexpected sites of metastases.

We reported a case of recurrent malignant phyllodes tumor of breast with multiple sites of unusual metastases detected by FDG PET/CT, highlighting the potential application of FDG PET/CT in the investigation of phyllodes tumor of breast. To date, there is no international consensus of role of FDG PET/CT in the management of malignant phyllodes tumor. With its well-known risk of metastases to almost all organs according to literatures, a whole-body assessment in high-risk cases seems to be necessary to detect metastases at the unexpected sites. FDG PET/CT may play a role, but further prospective study is required.

## Conclusion

We report a case of malignant phyllodes tumor of breast with recurrence after surgery and multiple unusual metastatic sites detected by FDG PET/CT. Despite the fact that there is no consensus of role and application of FDG PET/CT in phyllodes tumor of breast, it may play a role in the detection of unexpected distant metastasis for malignant phyllodes tumor with its high sensitivity of lesion detection and whole-body assessment in a single scan. Further prospective study is needed to evaluate the role of FDG PET/CT in the management of phyllode tumor of breast.
